# Case Report: Prenatal Diagnosis of a Fetus With Harlequin Ichthyosis Identifies Novel Compound Heterozygous Variants: A Case Report

**DOI:** 10.3389/fgene.2020.608196

**Published:** 2021-01-12

**Authors:** Jiao Liu, Xingyu Zhang, Weilan Wang, Xiaofang Lan, Minyue Dong, Kai Yan, Yongliang Lei, Penglong Chen, Mufeng Yang, Qunda Shan, Chunlei Jin

**Affiliations:** ^1^Center of Medical Prenatal Diagnosis, Lishui Maternity and Child Health Care Hospital, Lishui, China; ^2^Shanghai Children’s Medical Center, Institute of Pediatric Translational Medicine, Shanghai Jiao Tong University School of Medicine, Shanghai, China; ^3^Department of Dermatology, Shaoxing Central Hospital, Shaoxing, China; ^4^Department of Reproductive Genetics, Women’s Hospital, School of Medicine, Zhejiang University, Hangzhou, China

**Keywords:** harlequin ichthyosis, *ABCA12* gene mutation, skin abnormalities, fetus, autosomal recessive

## Abstract

**Background:**

Harlequin ichthyosis (HI) is the most severe form of the keratinizing disorders, and it is characterized by whole-body hard stratum corneum. *ABCA12* has been identified as the major disease-causing gene of HI.

**Methods:**

A case of HI was prenatally diagnosed by ultrasonography and genetic tests. The fetus had been found with dentofacial deformity and profound thickening of the palm and plantar soft tissues. Chromosomal microarray analysis (CMA) and whole exome sequencing (WES) were then performed on the amniotic fluid to identify germline pathogenic variants for the fetus. Candidate variants were verified by Sanger sequencing.

**Results:**

Compound heterozygous frameshift variants (p.Q719QfsX21; p.F2286LfsX6) of *ABCA12* were identified for the fetus, suggesting the former variants were maternally inherited and the latter paternally inherited. The fetus was terminated.

**Conclusion:**

A prenatal molecular diagnosis is an important approach for the prevention of HI. In the study, we provided a successful case of genetic counseling for a family with an HI baby.

## Introduction

Inherited ichthyoses are a group of genetic defects characterized by generalized dry skin, scaling, and hyperkeratosis. Harlequin ichthyosis (HI; OMIM 242500), the most severe form of ichthyoses, is a rare genodermatological disease. The clinical feature of HI is characterized by a markedly thickened stratum corneum covering the whole body, which cracks and forms erythematous fissures soon after birth. The skin abnormalities also affect the shape of eyelids, ears, nose, and lips (Thomas et al., 2006; Rajpopat et al., 2011).

HI is an autosomal recessive disease with an incidence of 1 in 3,00,000 births (Ahmed and O’Toole, 2014). Pathogenic variants of *ABCA12* [OMIM^∗^607800] have been demonstrated as the major causes of HI (Akiyama et al., 2005; Kelsell et al., 2005; Thomas et al., 2006). The *ABCA12* protein functions as a lipid transporter transferring lipids from the cytosol into lamellar granules in healthy skin. The lamellar granules fuse with the cell membrane and release their content into the intercellular lamellae. In the skin of HI patients, dysfunction of *ABCA12* results in disordered lipid transfer. As a consequence, abnormal lipid-containing vacuoles form in the corneocyte cytoplasm. The skin turns into a defective formation of the lipid layer, and the stratum corneum grows remarkably thickened (Hovnanian, 2005).

Here, we report novel compound heterozygous variants in a Chinese HI case through prenatal molecular diagnosis.

## Case Report

**FIGURE 1:**
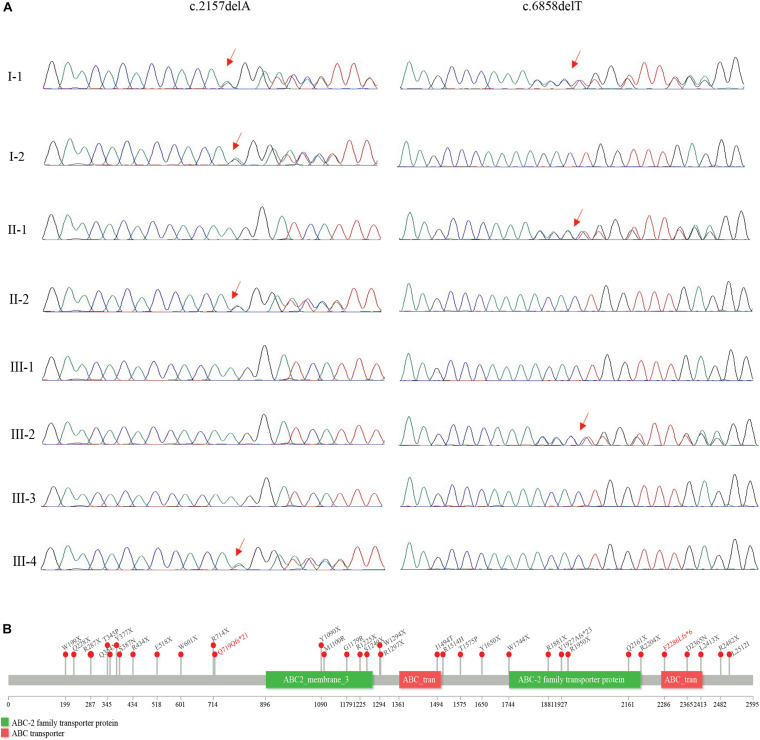
Clinical presentation of the proband. **(A)** Pedigree of the family. Transmission of the compound heterozygous variants of the HI baby were labeled in the three-generation family. **(B)** Clinical features of the HI baby. The fetus was covered in a thick, plate-like collodion membrane over the entire body surface at birth; the hands and feet in particular were enveloped by a wax-like cast of extremely tight skin. The features of this fetus were consistent with harlequin ichthyosis, displaying multiple malformations such as a short face, abnormal nasal bone, ear and mandible, and a cleft palate.

Genetic counseling was provided to a 29-year-old Chinese woman (gravida 5, para 1) during her gestational 23 weeks since she had abnormal fetal ultrasonography results. The woman delivered a healthy female baby at term during her first pregnancy (Figure 1A). Ultrasonography at the 12th week of this pregnancy indicated that the fetus had multiple malformations, such as a short face, abnormal nasal bone, ear and mandible, and a cleft palate. At the 16th week of pregnancy, the fetus showed profound thickening of the palm and plantar soft tissues according to ultrasonography. The clinical diagnosis was not determined based on ultrasonography. Subsequent genetic tests were performed to make a definite diagnosis for the fetus. Combined with the results of whole-exome sequencing (WES) and ultrasonography, the fetus was finally diagnosed as HI. Table 1 shows a timeline of the case report.

**TABLE 1:** Timeline of case report.

Gestational age	Summaries	Diagnostic testing	Interventions
12 weeks	Multiple malformations such as short face, abnormal nasal bone, ear and mandible, cleft palate.	Ultrasound examination	
12 weeks	No abnormality.	CNV array tests	Amniocentesis
16 weeks	No abnormality.	Karyotype Analysis	Amniocentesis
16 weeks	Profound thickening of the palm and plantar soft tissues.	Ultrasound examination	
16 weeks	Two heterozygous frameshift variants (p.Q719QfsX21; p.F2286LfsX6) of *ABCA12* were found in the fetus.	Whole exome sequencing	Amniocentesis
23 weeks	Diagnosed as HI.	Autopsy of the fetus	Rivanol-induced abortion

The baby was induced through vaginal delivery at 23 gestational weeks. The autopsy reports (Figure 1B) are as follows: weight, 1,990 g; body length, 42.5 cm; head circumference, 33 cm; chest circumference, 27 cm; abdomen circumference, 24 cm; no necropsy; medium nutrition; there was no obvious ectropion of eyelid and undeveloped eyeball; the distance between the two eyes was 3.5 cm on the inside and 7 cm on the outside; nose collapse, the nasal bone showed poor development; the mouth and lip were everted and edematous, fish-like, with protruding alveolar and short mandible; the development of bilateral auricles was poor and the auditory canal existed; the skin of the whole body was covered with light yellow horns (0.2 cm thick, armor-like); the hands and feet were poorly developed; the fingers and toes were all flexed and claw-like; the development of external genitalia was poor; the gender discrimination was unclear (male consideration), and there was an anus. The liver was 81.2 g, red soft, 10 × 6 × 3 cm in size, and it was located at 4 cm below the xiphoid process and 2.5 cm below the right costal margin; the heart was 12 g with a size of 4.5 × 3 × 2.5 cm with an unclosed foramen ovale, and the heart ventricle wall was 0.5 cm thick. The peripheral diameters of the tricuspid valve, pulmonary artery, and mitral valve were 4, 1.5, and 3 cm, respectively; and the sizes of the two kidneys were both 4 × 2 × 2 cm. The left lung with two lobes was 5.5 × 3 × 2 cm in size and 14.6 g in weight, and it was also clear and sink in water; the right lung with three lobes was 6 × 5 × 2.5 cm in size and 19.8 g in weight, and it was also clear and sink in water. Pathological examination of the skin indicated severe hyperkeratosis of the epidermis.

**FIGURE 2:**
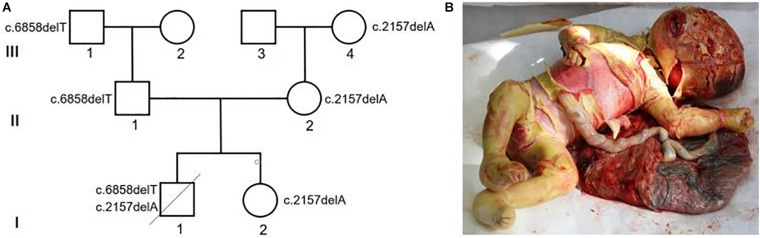
Verification of variants in the HI baby. **(A)** Sanger sequencing verification of the HI compound heterozygous variants identified by WES in the family. **(B)** Summarization of published HI variants. The variants identified in this study have been highlighted in red.

The amniotic fluid was collected for genetic analysis. Genomic DNA was extracted using the QIAmp DNA Blood Mini Kit (Qiagen, Hilden, Germany) with standard protocols. The CytoScan 450K array (Affymetrix, CA, United States) was used to detect copy number variations (CNV). No abnormalities were identified through CNV array tests and karyotype analysis, so the whole-exome sequencing (WES) was performed for all the family members (including the fetus’ sister, parents, and grandparents) to clarify the genetic risk for the next pregnancy. For WES, exome capture was performed using SureSelectXT Human All Exom V4 (Agilent Technologies, CA, United States). Sequencing was implemented on the Illumina HiSeq 2000 system (Illumina, CA, United States). Sequencing data analysis was performed following the GATK best practice. Detected variants were annotated and filtered using Annovar based on information of functional prediction (e.g., Polyphen2, SIFT, varianttaster, REVEL MuMetaSVM), disease association (e.g., ClinVar, OMIM, GWAS, HGMB, and SwissVar) and population allele frequency (e.g., dbSNP150, ExAC, 1000 genome phase3, EVS). A compound heterozygote for the variants c.2157delA (p.Q719QfsX21) and c.6858delT (p.F2286LfsX6) was identified. Both variants were frameshift deletions. The variants were verified using Sanger sequencing with a set of primers (*ABCA12*-17F: 5′-ATTATCAGGTTCTCTTTCTCTGTTG-3′, *ABCA12*-17R: 5′-CTATTTTTATTCTTGGGGAAAATT-3′; *ABCA12*-46F: 5′-GAGAGATACAAAAGCAATGTCTCA, *ABCA12*-46R: 5′-CTCATTTAAGTATGTTGTACTCGCT-3′). The results suggested that the sister, the mother, as well as the maternal grandmother carried the heterozygous c.2157delA, and the father and the paternal grandmother carried the heterozygous c.6858delT (Figures 1A, 2A).

## Discussion

The overall incidence of HI is 1 in 3,00,000 births (Layton, 2005) and the rate of recurrence is about 25% in subsequent pregnancies. HI can therefore largely be explained by genetic variations. With the development of medical techniques, HI with a family history could be successfully diagnosed at the prenatal stage (Yanagi et al., 2008; Ahmed and O’Toole, 2014; Rathore et al., 2015; Xie et al., 2016; Jian et al., 2018; Loo et al., 2018; Sheth et al., 2018; Liang et al., 2019). Here, we report a case without a family history of HI. Combining the information of ultrasonography and molecular diagnosis, we provided sufficient information on genetic counseling for the family.

More than 70 variants of *ABCA12*, accounting for autosomal recessive congenital ichthyoses, have been reported (Ahmed and O’Toole, 2014; Gurkan et al., 2015; Washio et al., 2017; Sheth et al., 2018). Pathogenic variants of the *ABCA12* gene cause ARCI 4A [OMIM:601227, including congenital ichthyosiform erythroderma (CIE) and lamellar ichthyosis (LI)] and 4B (HI, OMIM:242500). A previous analysis concerning genotype–phenotype correlation of *ABCA12* variants indicated that the CIE or LI phenotypes are often due to missense variants *ABCA12* (Loo et al., 2018). HI was suggested to be caused by truncation or deletion variant in a conserved region of *ABCA12*, which leads to a severe loss of ABCA12 protein function (Akiyama, 2006). In this fetus, the c.6858delT variant inherited from the father is predicted to create a frameshift starting at codon Phe2286, resulting in a truncated protein. It has been previously reported in a female infant with HI who carries a novel missense variant in the gene (Loo et al., 2018), and in a male patient with atypical ARCI who also carries a missense variant in the gene (Scott et al., 2013a). The c.2157delA variant inherited from the mother is predicted to create a frameshift starting at codon Gln719 (p.Q719QfsX21). This novel variant has not been reported previously and is not listed in any population database.

In this report, we summarized published HI variants (splicing variants were not showed) that altered the *ABCA12* protein sequence (Figure 2B). It is notable that most of these HI variants caused halt gain or frameshift, which should be damaging for the *ABCA12* function. Multiple genes, such as *ABCA12*, *NIPAL4*, *PNPLA1*, *LIPN*, *ST14*, *TGM1*, *ALOX12B*, *ALOXE*, and *CYP4F22*, have been known to be involved in congenital ichthyosis (Sheth et al., 2018). Among these genes, *ABCA12* has been characterized as the major disease-causing genes of HI (Thomas et al., 2006). *ABCA12* is a member of the adenosine triphosphate binding cassette (ABC) superfamily of active transporters (Uitto, 2005). Variants in ABC genes cause a variety of diseases such as cystic fibrosis, Tangier disease, pseudoxanthoma elasticum, Dubin-Johnson syndrome, and X-linked adrenoleukodystrophy (Hovnanian, 2005; Uitto, 2005). Dysfunction of the *ABCA12* protein can directly affect the formation of the intercellular lipid layers, which is essential for epidermal barrier function (Hovnanian, 2005; Scott et al., 2013b). Although most HI cases are caused by *ABCA12* variants, rare *ABCA12* variants are difficult to identify using low throughput methods such as Sanger sequencing since this gene contains 53 exons spanning more than 200 kb genomic region. Therefore, WES should be the most cost-effective method for rare variant of HI at present.

The HI fetuses usually had a fatal outcome during the early neonatal period, usually within the first 2 weeks (Layton, 2005). In the past 20 years, the prognosis of HI infants has improved with advances in neonatal intensive care and retinoid therapy. However, the quality of life of HI patients is seriously affected in the long term. In the present study, the family chose to terminate the pregnancy after genetic counseling. The prenatal ultrasound diagnosis of HI was confirmed by molecular diagnosis and skin biopsy. Through WES, we identified two inherited heterozygous frameshift variants that would be helpful for the diagnosis of HI in the future.

## Patient’s Perspective

The fetus was terminated at the 23rd gestational week according to the molecular diagnosis report. Although depressed, the mother was grateful to the genetic consultant for the genetic tests since she could avoid having a baby with HI in the future.

## Data Availability Statement

The original contributions presented in the study are included in the article/Supplementary Material, further inquiries can be directed to the corresponding author/s.

## Ethics Statement

The studies involving human participants were reviewed and approved by the Ethics Committee of Lishui Maternity and Child Health Care Hospital. Written informed consent to participate in this study was provided by the participants’ legal guardian/next of kin. Written informed consent was obtained from the minor(s)’ legal guardian/next of kin for the publication of any potentially identifiable images or data included in this article.

## Author Contributions

All authors listed have made a substantial, direct and intellectual contribution to the work, and approved it for publication.

## Conflict of Interest

The authors declare that the research was conducted in the absence of any commercial or financial relationships that could be construed as a potential conflict of interest.
